# Mr. Stephen Luther Martin

**Published:** 1910-08-06

**Authors:** 


Mr. Stephen Luther Martin, of Oxford Terrace, Hyde Park, has died in London. He took his L.S.A. in 1892, having been a student at the London Hospital.

